# Participation of polymorphonuclear leukocyte-derived factor in murine tumour cell killing.

**DOI:** 10.1038/bjc.1985.229

**Published:** 1985-10

**Authors:** M. Ikenami, M. Yamazaki

## Abstract

Previous studies showed that murine polymorphonuclear leukocytes (PMNs) lyze tumour cells in the presence of wheat germ agglutinin or actinomycin D. This paper reports studies on whether a soluble factor participates in PMN-mediated cytolysis dependent on lectin or a chemotherapeutic drug. Tumour lysis was observed with supernatants from PMN cocultured with wheat germ agglutinin or actinomycin D. The supernatant from cultures of PMNs alone was not cytotoxic, but addition of these agents to the supernatant induced tumour lysis. PMNs released a soluble factor spontaneously into the medium and cytolysis was induced by a combination of this factor and wheat germ agglutinin or actinomycin D. This factor was not an oxygen metabolite, but a protein with a molecular weight of approximately 100 K daltons. These results suggest that a soluble factor(s) from PMNs participates in tumour killing in cooperation with appropriate reagents.


*Br. J. Cancer* (1985), **52**, 575–581

### Participation of polymorphonuclear leukocyte-derived factor in murine tumour cell killing

M. Ikenami & M. Yamazaki

*Faculty of Pharmaceutical Sciences, Teikyo University, Sagamiko, Tsukui-gun Kanagawa 199-01, Japan*

**Summary** Previous studies showed that murine polymorphonuclear leukocytes (PMNs) lyze tumour cells in the presence of wheat germ agglutinin or actinomycin D. This paper reports studies on whether a soluble factor participates in PMN-mediated cytolysis dependent on lectin or a chemotherapeutic drug. Tumour lysis was observed with supernatants from PMN cocultured with wheat germ agglutinin or actinomycin D. The supernatant from cultures of PMNs alone was not cytotoxic, but addition of these agents to the supernatant induced tumour lysis. PMNs released a soluble factor spontaneously into the medium and cytolysis was induced by a combination of this factor and wheat germ agglutinin or actinomycin D. This factor was not an oxygen metabolite, but a protein with a molecular weight of ~100K daltons. These results suggest that a soluble factor(s) from PMNs participates in tumour killing in cooperation with appropriate reagents.

Polymorphonuclear leukocytes (PMNs) are important in defence against infection and are also found in histological sections of neoplasms and in cytological preparations of malignant tissues (Dvorak *et al.*, 1978; Godleski *et al.*, 1970; Slauson *et al.*, 1975). PMNs are cytotoxic *in vitro* to tumour cells of animals (Fisher *et al.*, 1979; Pickaver *et al.*, 1972; Lichtenstein & Kahle, 1985) and humans (Chee *et al.*, 1978; Gerrard *et al.*, 1981; Korec *et al.*, 1980; Takasugi *et al.*, 1975; Clark & Klebanoff, 1979).

Previously, we showed that PMNs from the peritoneal cavity of mice could kill murine tumour cells *in vitro* on addition of appropriate mediators; *viz* plant lectins (Ikenami & Yamazaki, 1983), animal lectins (Yamazaki *et al.*, 1983) antitumour antibody (Tsunawaki *et al.*, 1983), anticancer chemotherapeutic drugs (Ikenami *et al.*, 1985) and immunomodulators (Morikawa *et al.*, 1985). Reactive oxygen species produced by PMNs are important in the lytic process by immunomodulators (Morikawa *et al.*, 1985), but the mechanisms of other types of killing are unknown. In this work, we investigated the mechanisms of PMN-mediated cytolysis dependent on lectin and chemotherapeutic drug, by studies on whether soluble factor from PMNs participates in tumour killing *in vitro*. We found that a PMN-derived factor can lyse tumour cells in cooperation with wheat germ agglutinin or actinomycin D and that this factor is a protein of high molecular weight.

### Materials and methods

### *Mice*

Inbred male C3H/He and DDY mice were obtained from Shizuoka Experimental Animal Farm (Shizuoka, Japan). Mice were used at 8–11 weeks of age.

### *Tumour cells*

MM46, a transplatable ascites tumour from a spontaneous mammary adenocarcinoma in a C3H/He mouse, was mainly used as a target cell. MM48 mammary adenocarcinoma and MH134 hepatoma cells were also used as target cells. L929 cells were harvested from *in vitro* culture.

### *Polymorphonuclear leukocytes (PMNs)*

Cells were prepared as described previously (Tsunawaki *et al.*, 1983). Briefly, 2 ml of 12% casein solution was injected into the peritoneal cavity of mice and the peritoneal exudate was harvested 6 h later, passed through nylon mesh and centrifuged at 300 *g* for 5 min. The precipitated cells were washed twice with RPMI-1640 medium (Nissui Seiyaku Co., Tokyo) supplemented with 100 U ml$^{-1}$ of penicillin (Banyu Pharmaceutical Co., Tokyo) and 100 $\mu$g ml$^{-1}$ of streptomycin (Meiji Seika Co., Tokyo). Usually, the peritoneal cells were suspended in RPMI-1640 medium containing 5% heat-inactivated foetal calf serum (Gibco, Grand Island, NY; called medium hereafter). They were stained with Giemsa stain and the proportions of PMNs were determined by morphological observation. These peritoneal cells, containing 93–98% of polymorphonuclear leukocytes, were used as the polymorphonuclear leukocyte preparation. About $10^8$ PMNs were obtained from a C3H/He mouse.

### *Cytolytic assay*

Cytolysis of MM46 tumour cells was assayed as described previously (Yamazaki *et al.*, 1975). Briefly,

Correspondence: M. Yamazaki.
Received 22 April 1985; in revised form 22 June 1985.

© The Macmillan Press Ltd., 1985

PMN-culture supernatants and $^{51}$Cr-labelled MM46 tumour cells ($5 \times 10^3$ cells) were mixed in wells (7 mm diameter) of flat-bottomed microplates. The mixture were incubated in 0.2 ml of medium for 18–24 h at 37°C under 5% $CO_2$ in air and the radioactivity of the supernatant was measured. Cytolytic activity was calculated as follows:

$$\text{Cytolysis (\%)} = \frac{\text{Experimental count} - \text{control count}}{\text{Maximum releasable count} - \text{control count}} \times 100$$

Maximum release of $^{51}$Cr was measured by freezing-thawing labelled tumour cells 3 times. The control count was measured as the radioactivity released from labelled cells in the presence of wheat germ agglutinin or actinomycin D without culture supernatant. The control count was usually equivalent to the count released spontaneously from labelled cells alone.

Cytolysis of L929 cells was measured by the method of Ruff & Gifford (1980). Briefly, L929 cells ($8 \times 10^4$ cells) and PMN-culture supernatants were mixed in the wells (7 mm diameter) of flat-bottomed microplates, and incubated in a $CO_2$ incubator for 18 h. Then, the medium was removed and residual cells were stained for 15 min with crystal violet. After addition of 0.1 ml of sodium dodecyl sulfate (0.5%), absorbance at 590 nm of the supernatant was measured in a photometer (Myreader 7, Sanko Junyaku Co., Ltd., Tokyo). Cytolytic activity was calculated as follows:

$$\text{Cytolysis (\%)} = \left(1 - \frac{\text{Experimental absorbance}}{\text{Control absorbance}}\right) \times 100$$

*Cytolysis in Marbrook vessels*

Marbrook vessels with 2 chambers separated by a nuclepore membrane (Pore size, 0.4 $\mu$m; thickness 10 $\mu$m; Nuclepore Co., Pleasanton, CA) were prepared. PMNs ($1.2 \times 10^7$) were introduced into the outer chamber, and $3 \times 10^4$ $^{51}$Cr-labelled MM46 tumour cells were into the inner chamber. The cells were incubated in 2 ml of medium with or without wheat germ agglutinin (30 $\mu$g ml$^{-1}$) at 37°C for 24 h. As controls, $^{51}$Cr-labelled MM46 tumour cells were placed in the outer chamber with PMNs. After incubation, the radioactivities of the supernatants of the inner and outer chambers were measured.

*Reagents*

Wheat germ agglutinin was purchased from E-Y Laboratories (San Mateo, CA). Actinomycin D, catalase, arginine, trypsin and soybean trypsin inhibitor were obtained from Sigma Chemical Co., (St. Louis, MO). Leupeptin and bestatin were gifts from Dr T. Takeuchi (Institute of Microbiol Chemistry, Tokyo).

*Gel filtration*

The PMN-culture supernatant was applied to a column of Sephacryl S-300 (Pharmacia Fine Chemicals, Sweden) previously equilibrated with phosphate-buffered saline (pH 7.4) and material was eluted with the same buffer. Fractions of 10 ml were collected and their cytolytic activity was tested in the presence of wheat germ agglutinin or actinomycin D.

### Results

*Cytolytic activity of supernatants of cultures of PMNs*

We used Marbrook vessels to study the role cell-to-cell interaction in cytolysis, i.e. whether effector-target cell interaction is necessary for cytolysis. When effector PMNs and target tumour cells were incubated separately in different chambers, cytolysis was observed in the presence of wheat germ agglutinin just as when both types of cells were incubated in the same chamber (Figure 1). When

**Figure 1** Wheat germ agglutinin-dependent PMN-mediated tumour lysis in Marbrook vessels. *MM46; $^{51}$Cr labelled MM46 tumour cells, MM46: intact MM46 tumour cells. WGA: wheat germ agglutinin. Bars. indicate sd ($n = 3$).

unlabelled tumour cells were killed by PMNs in the outer chamber in the presence of wheat germ agglutinin, $^{51}$Cr-labelled tumour cells in the inner chamber were also killed.

Similar results were obtained in actinomycin D-dependent PMN-mediated cytolysis: cell-free supernatants from cocultures of PMNs with actinomycin D lysed tumour cells (Figure 2).

These results suggested that direct contact between effector PMNs and target tumour cells was not essential for tumour lysis and that the lysis involved a soluble factor(s) released into the medium.

**Figure 2** Cytolytic activity of culture medium. PMNs ($1\times10^7$ cells) or MM46 tumour cells ($3\times10^5$ cells) were cultured for 1 h with or without actinomycin D ($5\,\mu g\,ml^{-1}$). Medium containing 50% culture supernatant and $^{51}$Cr labelled MM46 tumour cells ($5\times10^3$ cells) was incubated for 18 h. Bar indicates sd ($n=3$).

Figure 3 shows the dose-response curves of culture supernatants from PMNs with wheat germ agglutinin and actinomycin D respectively. Cytolytic activities were detected with up to 4-fold dilutions of both supernatants.

### *Characterization of supernatants from cultures of PMNs*

The specificity of cytolysis was examined with several kinds of target cells. Table I shows that 3 other syngeneic tumour cells, MM48, MH134 and L929 cells, were lyzed by the supernatant of cultures of PMNs with wheat germ agglutinin. However, no cytolysis of normal spleen cells was observed.

Next, we examined whether proteases, arginase and oxygen metabolites act as lytic substances in cytolysis by the supernatants. For examination of the effects of proteases the following inhibitors were used: soybean trypsin inhibitor against trypsin, leupeptin against plasmin, trypsin and papain, and bestatin against aminopeptidase B and leucine aminopeptidase. For examination of the effects of oxygen metabolities, such as $H_2O_2$, catalase was used as a scavenger of these substances. As shown in Table II, protease inhibitors, oxygen scavenger and arginine did not inhibit the cytolytic activities of either supernatant.

Next, we examined the effects of heat- and trypsin-treatments on the cytolytic activities of

**Figure 3** Cytolytic activity of supernatants from PMN-coculture with lectin or actinomycin D. (a) PMNs ($2\times10^7$ cells) were cultures with (△) or without (▲) actinomycin D ($0.5\,\mu g\,ml^{-1}$) for 3 h. Medium containing the indicated concentration of culture supernatant and L929 cells ($8\times10^4$ cells) was incubated for 18 h. (b) PMNs ($2\times10^7$ cells) were cultured with (○) or without (●) wheat germ agglutinin ($100\,\mu g\,ml^{-1}$) for 3 h. Medium containing the indicated concentration of culture supernatant and L929 cells ($8\times10^4$ cells) was incubated for 18 h. Bars indicate sd ($n=3$).

**Table I** Target specificity of supernatants from PMN cultures

| | *Cytolysis (%) of target cells*[b] | | | | |
|---|---|---|---|---|---|
| *Supernatant*[b] *from* | *MM46* | *MM48* | *MH134* | *L929* | *Spleen cells* |
| PMN culture | 0±1 | 0±1 | 10±5 | 13±2 | −1 |
| PMN coculture with wheat germ agglutinin | 56±11 | 42±4 | 27±9 | 60±7 | −3 |

[a]Supernatants were obtained after 5 h cultures of PMNs with or without wheat germ agglutinin (50 μg ml$^{-1}$); [b]Cytolysis of tumour cells was assayed by $^{51}Cr$ release method. Cytolysis of spleen cells was determined by the dye exclusion test. Cytolysis (35%) by medium alone was subtracted as a background value. Mean ± s.d. ($n=3$).

**Table II** Effects of various treatments on cytolytic activities of culture supernatants

| | *Cytolysis (%) by supernatant*[b] *with* | | | |
|---|---|---|---|---|
| | *actinomycin D* | | *wheat germ agglutinin* | |
| *Treatment*[a] | *before treatment* | *after treatment* | *before treatment* | *after treatment* |
| Protease inhibitor | | | | |
| soybean trypsin inhibitor (1 mg ml$^{-1}$) | 21±5 | 21±3 | 19±3 | 19±3 |
| leupeptin (125 μg ml$^{-1}$) | N.D. | | 17±1 | 22±5 |
| bestatin (125 μg ml$^{-1}$) | N.D. | | 17±1 | 16±2 |
| Catalase (1000 U ml$^{-1}$) | 26±7 | 23±2 | ND | |
| (2000 U ml$^{-1}$) | N.D. | | 17±3 | 16±2 |
| Arginie (500 μg ml$^{-1}$) | 31±2 | 25±6 | 19±3 | 16±2 |
| Trypsin (250 μg ml$^{-1}$) | 19±2 | 2±1* | 11±1 | 4±1* |
| (500 μg ml$^{-1}$) | 19±2 | 0±4* | ND | |
| Heating (56°, 30′) | 21±2 | 5±3* | 17±1 | 13±2* |
| (70°, 60′) | 21±2 | 0±1* | 17±1 | 0±3* |

[a]Various inhibitors were added to the culture supernatant before its cytolytic activity was assayed; [b]Supernatants were obtained after 5 h-cocultures of PMNs with actinomycin D (0.5 μg ml$^{-1}$) or wheat germ agglutinin (100 μg ml$^{-1}$); * = significant difference ($P<0.05$).

supernatants. Table II shows that the cytolytic activity was lost on heating at 70° for 1 h and on trypsin treatment.

### *Spontaneous release of the soluble factor from PMNs*

As described above, supernatants from PMNs cocultured with wheat germ agglutinin or actinomycin D were cytolytic, but supernatants from PMNs cultured alone were not. Next, we examined whether these reagents were required to induce a factor from PMNs i.e., whether addition of these reagents to supernatants from PMNs cultured alone induced tumour lysis as well as supernatants from PMNs cocultured with these reagents.

As shown in Table III, tumour lysis was induced by addition of wheat germ agglutinin or actinomycin D to supernatants of PMN cultures. The supernatant alone was not cytolytic to tumour cells. Thus, cytolysis seemed to be induced by combination of a factor from PMNs and these reagents, and PMNs seemed to release this factor spontaneously into the medium.

The kinetics of release of this factor is shown in Figure 4. Maximum release was observed within 5 h, and no cytolytic activity was detected in supernatants of overnight cultures. Therefore, this factor may be released spontaneously from fresh PMNs but not from damaged or dead PMNs.

Next, the nature of the factor that induced cytolysis in cooperation with wheat germ agglutinin or actinomycin D was examined by subjecting the

**Table III** Cytolytic activities of supernatants from PMN-cultures

| *Culture*[a] | *Addition of drug in assay*[b] | *Cytolysis*[c] (%) |
|---|---|---|
| PMNs + actinomycin D | None | 17±4 |
| PMNs alone | Actinomycin D | 22±1 |
| PMNs alone | None | 3±3 |
| PMNs + wheat germ agglutinin | None | 22±4 |
| PMNs alone | Wheat germ agglutinin | 20±6 |
| PMNs alone | None | 0±5 |

[a]Supernatants were obtained from cultures of PMNs with or without drugs for 5 h; [b]Actinomycin D ($1 \mu g ml^{-1}$) or wheat germ agglutinin ($50 \mu g ml^{-1}$) was added to the supernantant of PMNs cultured alone and then cytolysis was assayed; [c]Cytolysis of MM46 tumour cells was assayed. Mean ± sd ($n=3$).

**Figure 4** Time course of spontaneous release of PMN-factor. PMNs ($2 \times 10^7$ cells) were cultured alone in PBS (1 ml) and supernatants were obtained at the indicated times. Medium containing 50% of PMN-culture supernatant and $^{51}Cr$-labelled MM46 tumour cells ($5 \times 10^3$ cells) was incubated for 24 h with actinomycin D ($0.3 \mu g ml^{-1}$) (△), or wheat germ agglutinin ($50 \mu g ml^{-1}$) (○). Bars indicate sd ($n=3$).

supernatants from serum free-cultures of PMNs to gel filtration on Sephacryl S-300. As shown in Figure 5, the cytolytic activity was recovered in a fraction corresponding to a mol. wt of about ~100 K daltons.

### Discussion

Previously we observed mediator-dependent cytolysis when murine syngeneic tumour cells were lyzed by casein-induced peritoneal PMNs in the

**Figure 5** Gel filtration of PMN-culture supernatants. PMNs ($2 \times 10^7$ cells $ml^{-1}$) were cultured for 5 h in 23 ml PBS. Concentrated supernatants (3 ml) were applied to a Sephacryl S-300 column (1.8 × 100 cm) and fraction of 10 ml were collected. Medium containing 50% of eluant and L929 cells ($8 \times 10^4$ cells) were incubated for 18 h in the presence of actinomycin D ($0.25 \mu g ml^{-1}$) (△), or wheat germ agglutinin ($25 \mu g ml^{-1}$) (○). (●) Absorbance at 280 nm. Marker proteins such as ferritin (440 K daltons), aldolase (158 K daltons) and ovalbumin (43 K daltons) were previously chromatographed on the same column.

presence of lectins (Ikenami & Yamazaki, 1983: Tsunawaki *et al.*, 1983; Yamazaki *et al.*, 1983), anti-cancer chemotherapeutic drugs (Ikenami *et al.*, 1985) or immunomodulators (Morikawa *et al.*, 1985*a*). In the present work, we examined the mechanisms of lectin- drugs-dependent PMN-mediated cytolysis. We found that supernatants from PMNs cocultured with wheat germ agglutinin or actinomycin D could lyse tumour cells (Figure 1 and 2). As far as we know, tumour lysis by the supernatant of PMN cultures has not been reported previously. We also observed tumour lysis on addition of wheat germ agglutinin or actinomycin D to the supernatant of PMNs cultured alone (Table III). However, the factor itself was not cytolytic to tumour cells. Thus, cytolysis seemed to be induced by the action of the factor with these reagents.

In our system, contamination with macrophages was slight (<3%) and purified PMNs (99.1–99.5%) also showed cytolytic activity in the presence of actinomycin D (Ikenami *et al.*, 1985) and wheat germ agglutinin (Tsunawaki *et al.*, 1983). Moreover, this factor was released spontaneously from glass-nonadherent cells, but not from glass-adheremt macrophage-rich cells, and PMNs could not lyse tumour cells in the presence of lipopolysaccharide, which is known to activate macrophages (data not shown). Therefore, the PMN-mediated cytolysis was not due to contaminating macrophages.

Most previous studies on PMN cytotoxicity have focussed on oxygen metabolities. PMNs have been shown to kill tumour cells through oxygen-dependent pathways (Clark & Klebanoff, 1979: Nathan *et al.*, 1979; Dallegri *et al.*, 1983). We also reported that hydrogen peroxide was an effector molecule in immunomodulator-dependent PMN-mediated tumour lysis (Morikawa *et al.*, 1985*b*). However, the present factor was different from oxygen metabolites such as hydrogen peroxide (Table II). This factor seemed to have a mol. wt of about ~100 K daltons (Figure 5), and to be a protein, since it was heat-labile and inactivated by trypsin (Table II). The activity was not inhibited by protease inhibitors and arginine. These data suggest that the factor is neither protease nor arginase, which are known to be effector molecules of macrophages. A lysosomal cationic protein from PMNs that induces cytolysis has been reported (Thorne *et al.*, 1984), but this seemed to differ from our factor in *mol. wt* and its direct cytotoxicity on target cells.

This factor was released spontaneously from fresh PMNs (Figure 4). From this finding and the fact that PMN-mediated cytolysis was inhibited by an inhibitor of protein synthesis, cycloheximide (data not shown), we conclude that damaged or dead PMNs do not release the factor, but rather that PMNs may die after production of this factor. Spontaneous productions of cytotoxins by alveolar macrophages (Sone *et al.*, 1984) and a macrophage-like cell line (Kull & Cuatrecases, 1984) have been reported. Our results suggest that this may also be the case with PMNs: exudate PMNs may secret a factor that participates in target killing at an inflammatory site *in vivo*. In fact, recently we found a similar soluble factor to that reported here in inflammatory ascites containing many PMNs (manuscript in preparation). At present the primary action of this factor on target cells and its synergistic actions with wheat germ agglutinin and actinomycin D are not clear. We are now purifying and characterizing the factor further.

### References

CHEE, D.O., TOWNSEND, C.M., Jr., GALBRAITH, M.A., EIBER, F.R. & MORTON, D.L. (1978). Selective reduction of human tumor cell population by human granulocytes *in vitro*. *Cancer Res.*, **38**, 4534.

CLARK, R.A. & KLEBANOFF, S.J. (1979). Role of the myeloperoxidase-$H_2O_2$-halide system in concanavalin A-induced tumor cell killing by human neutrophils. *J. Immunol.*, **122**, 2605.

DALLEGRI, F., FRUMENTO, G. & PATRONE, F. (1983). Mechanisms of tumor cell destruction by PMA-activated human neutrophils. *Immunology*, **48**, 273.

DVORAK, A.M., CONNELL, A.B., PROPPE, K. & DVORAK, H.F. (1978). Immunologic rejection of mammary adenocarcinoma (TA3-St) in C57BL/6 mice: Participation of neutrophils and activated macrophages with fibrin formation. *J. Immunol.*, **120**, 1240.

FISHER, B. & SAFFER, E.A. (1978). Tumor cell cytotoxicity by granulocytes from peripheral blood of tumor-bearing mice. *J. Natl Cancer Inst.*, **60**, 687.

GERRARD, T.L., COHEN, D.J. & KAPLAN, A.M. (1981). Human neutrophil-mediated cytotoxicity to tumor cells. *J. Natl Cancer Inst.*, **66**, 483.

GOLDESKI, J.J., LEE, R.E. & LEIGHTON, J. (1970). Studies on the role of polymorphonuclear leukocytes in neoplastic disease with chick embryo and Walker carcinosarcoma 256 *in vivo* and *in vitro*. *Cancer Res.*, **30**, 1986.

IKENAMI, M., MIZUNO, D. & YAMAZAKI, M. (1985). Drug-dependent cellular cytotoxicity mediated by polymorphonuclear leukocytes. *Gann*, (in press).

IKENAMI, M. & YAMAZAKI, M. (1983). Plant lectin-dependent polymorphonuclear leukocyte-mediated cytolysis. *J. Pharm. Soc. Japan (Yakugaku Zasshi)*, **103**, 1298.

KOREC, S., HERBERMAN, R.B., DEAN, J.H. & CANNON, G.B. (1980). Cytostasis of tumor cell lines by human granulocytes. *Cell. Immunol.*, **53**, 104.

KULL, C. Jr. & CUATRECASAS, P. (1984). Necrosin: Purification and properties of a cytotoxin derived from a murine macrophage-like cell line. *Proc. Natl Acad. Sci.*, (USA), **81**, 7932.

LICHTENSTEIN, A. & KAHLE, J. (1985). Anti-tumor effect of inflammatory neutriphils: Characteristics of *in vivo* generation and *in vitro* tumor cell lysis. *Int. J. Cancer.*, **35**, 121.

MORIKAWA, K., TAKEDA, R., YAMAZAKI, M. & MIZUNO, D. (1985*a*). Induction of tumoricidal activity of polymorphonuclear leukocytes by a linear β-1, 3-D-glucan and some other immunomodulators. *Cancer Res.*, **45**, 1496.

MORIKAWA, K., KAMEGAYA, S., YAMAZAKI, M. & MIZUNO, D. (1985*b*). Hydrogen peroxide as a tumoricidal mediator of polymorphonuclear leukocytes induced by a linear β-1, 3-D-glucan and some other immunomodulators. *Cancer Res.*, (in press.)

NATHAN, C.F., SILVERSTEIN, S.C., BRUKNER, L.H. & COHN, Z.A. (1979). Extracellular cytolysis by activated macrophages and granulocytes. II. Hydrogen peroxide as a mediator of cytotoxicity. *J. Exp. Med.*, **149**, 100.

PICKAVER, A.H., RATCLIFFE, N.A., WILLIAMS, A.E. & SMITH, H. (1972). Cytotoxic effects of peritoneal neutrophils on a syngeneic rat tumor. *Nature New Biol.*, **235**, 186.

RUFF, M.R. & GIFFORD, E. (1980). Purification and physicochemical characterization of rabbit tumor necrosis factor. *J. Immunol.*, **125**, 1671.

SLAUSON, D.O., OSBURN, B.I., SHIFRINE, M. & DUNGWORTH, D.L. (1975). Regression of feline sarcoma virus-induced sarcoma in dogs. 1. Morphologic investigations. *J. Natl Cancer Inst.*, **54**, 361.

SONE, S., TACHIBANA, K., ISHII, K., OGAWA, M. & TSUBURA, E. (1984). Production of a tumor cytolytic factor(s) by activated human alveolar macrophages and its action. *Cancer Res.*, **44**, 646.

TAKASUGI, M., AKIRA, D. & KINOSHITA, K. (1975). Granulocytes as effectors in cell-mediated cytotoxicity of adherent target cells. *Cancer Res.*, **35**, 2169.

THORNE, K.J.I., NORMAN, J.M., HAYDOCK, S.F., LAMMAS, D.A. & DUFFUS, P.H. (1984). Antibody-dependent cell-mediated cytotoxicity against IBR-infected bovine kidney cells by ruminant neutrophils: the role of lysosomal cationic protein. *Immunology*, **53**, 275.

TSUNAWAKI, S., OSHIMA, H., MIZUNO, D. & YAMAZAKI, M. (1983). Induction of polymorphonuclear leukocyte-mediated cytolysis by wheat germ agglutinin and antitumor antibody. *Gann*, **74**, 264.

YAMAZAKI, M., IKENAMI, M., KOMANO, H., TSUNAWAKI, S., KAMIYA, H., NATORI, S. & MIZUNO, D. (1983). Polymorphonuclear leukocyte-mediated cytolysis induced by animal lectin. *Gann*, **74**, 576.

YAMAZAKI, M., SHINODA, H. & MIZUNO, D. (1975). Co-operation between macrophages and a factor from lymphocytes in tumor lysis *in vitro*. *Gann*, **66**, 489.


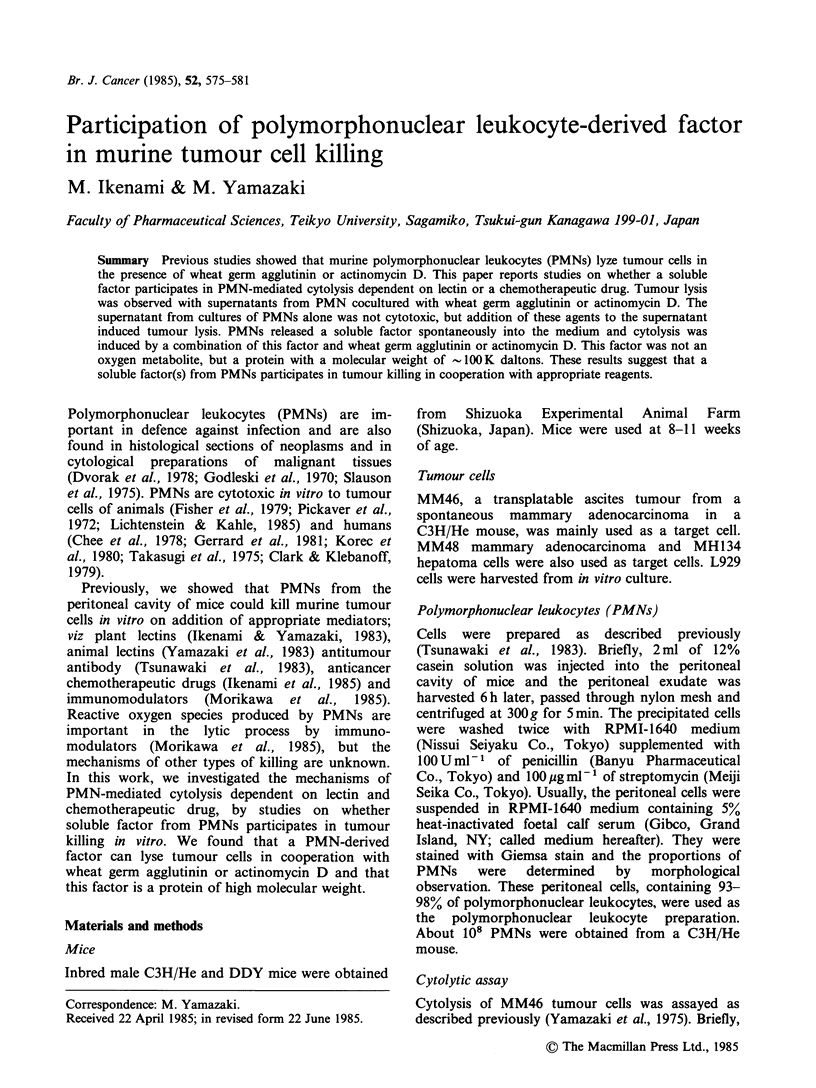

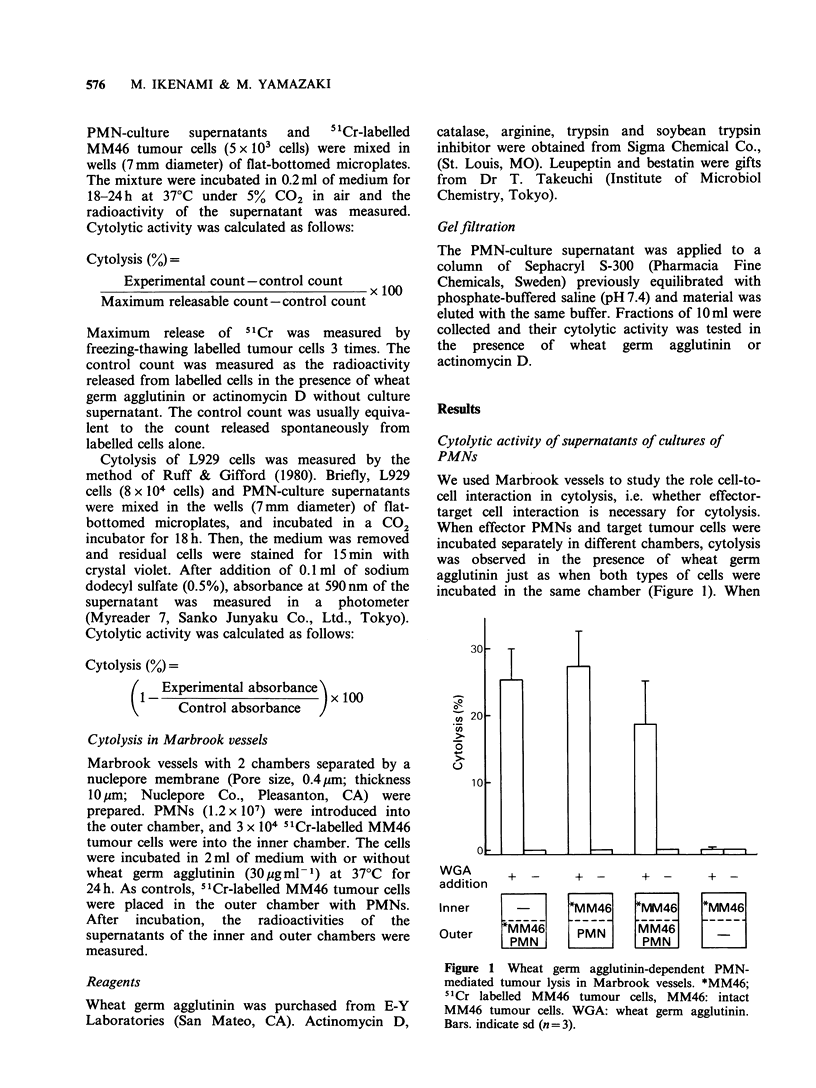

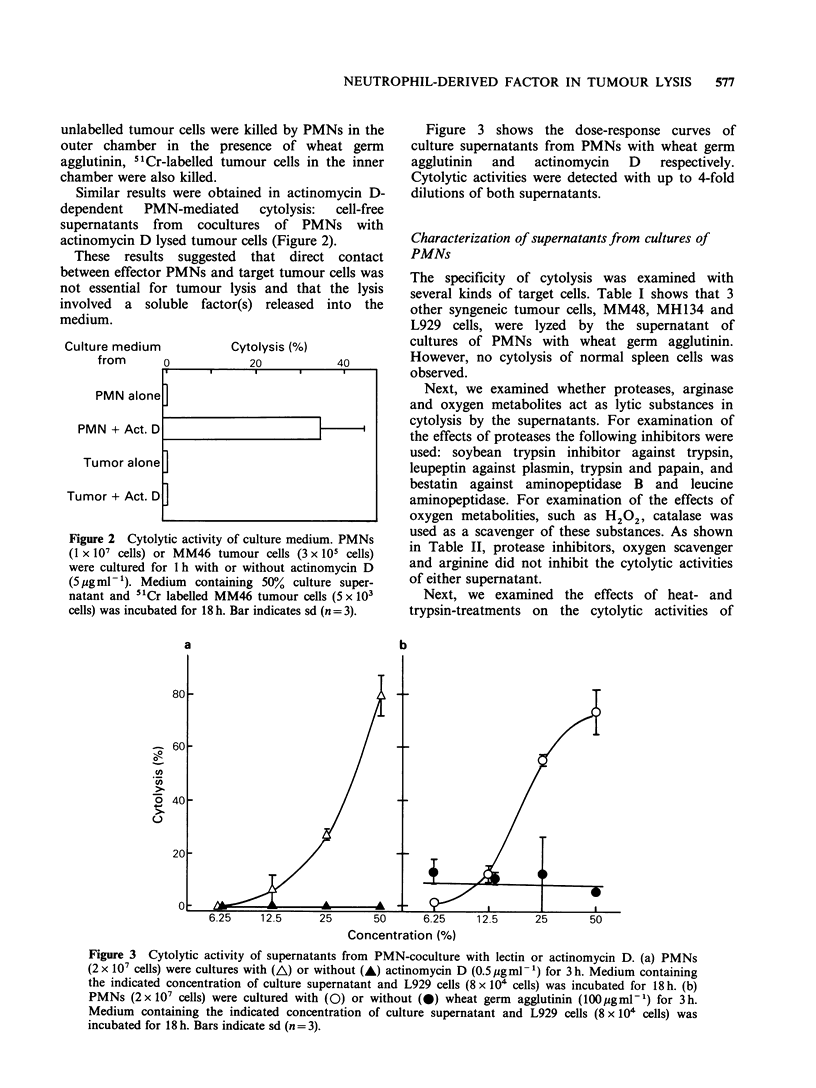

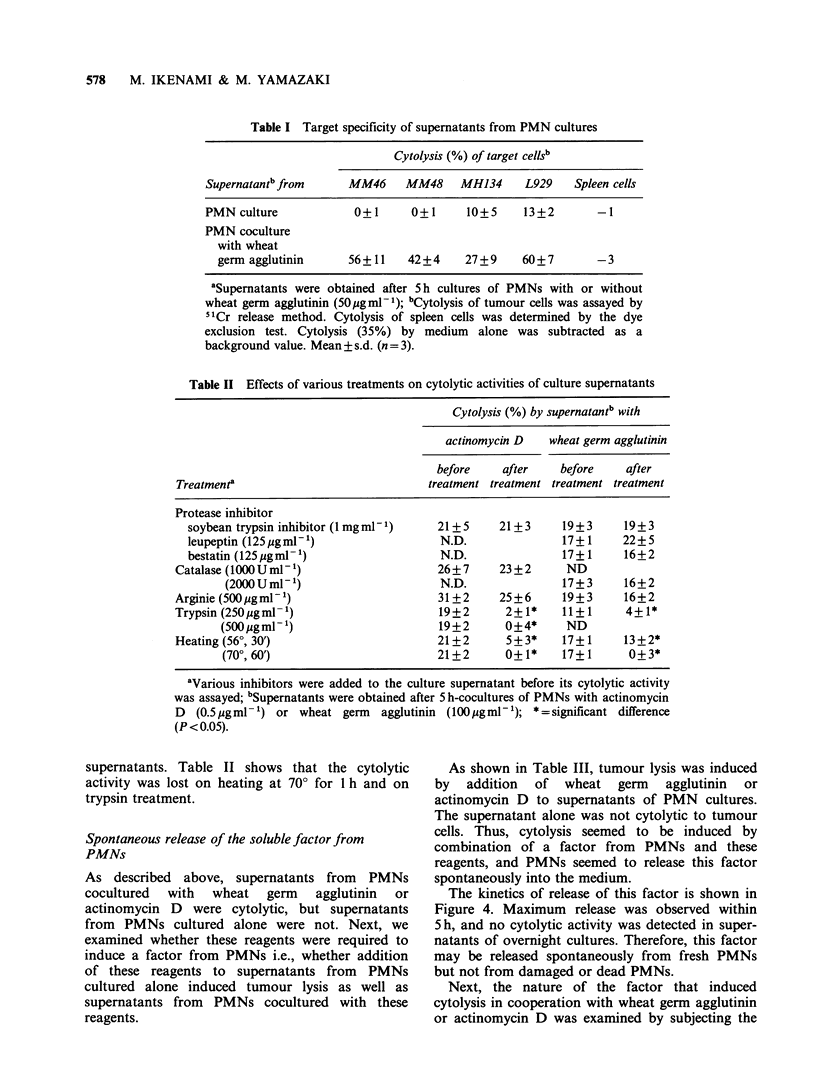

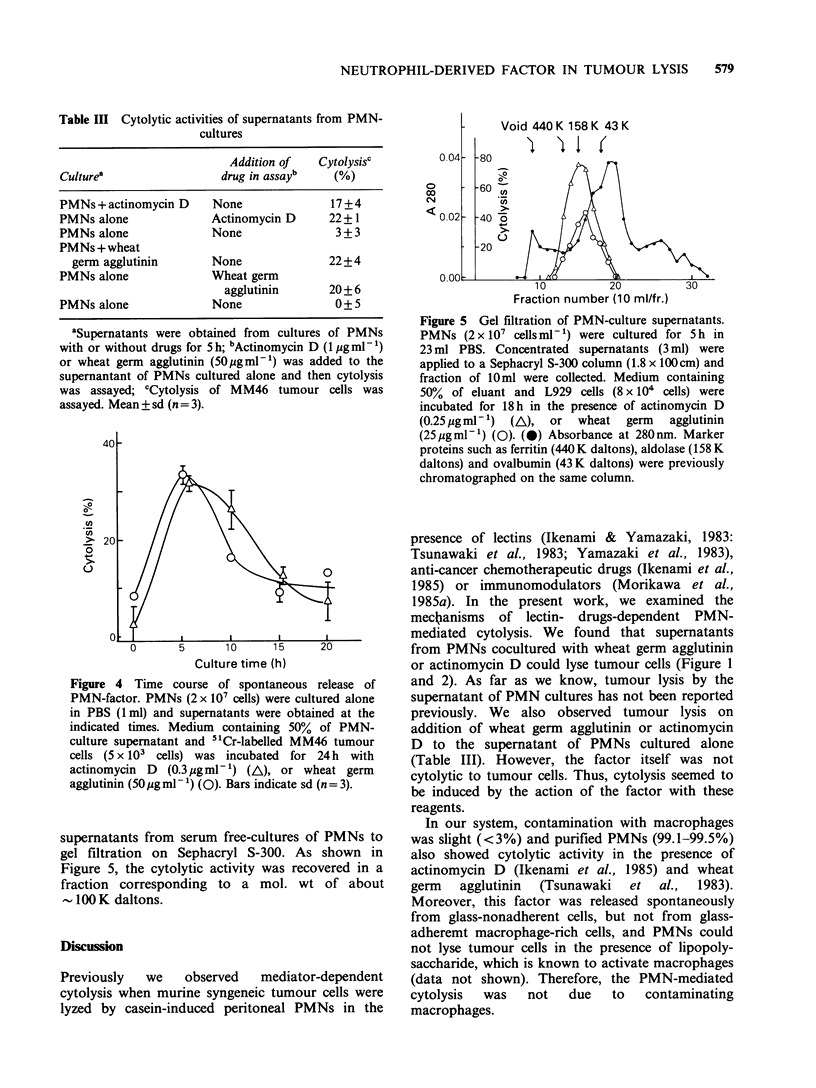

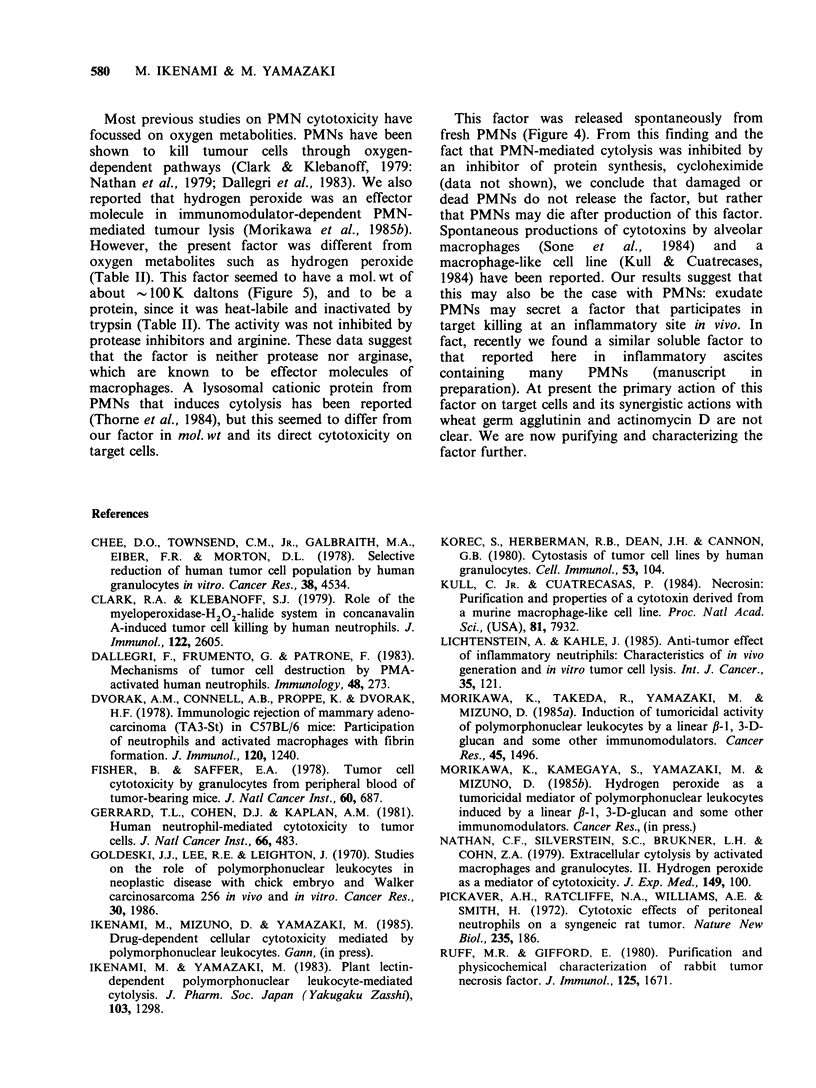

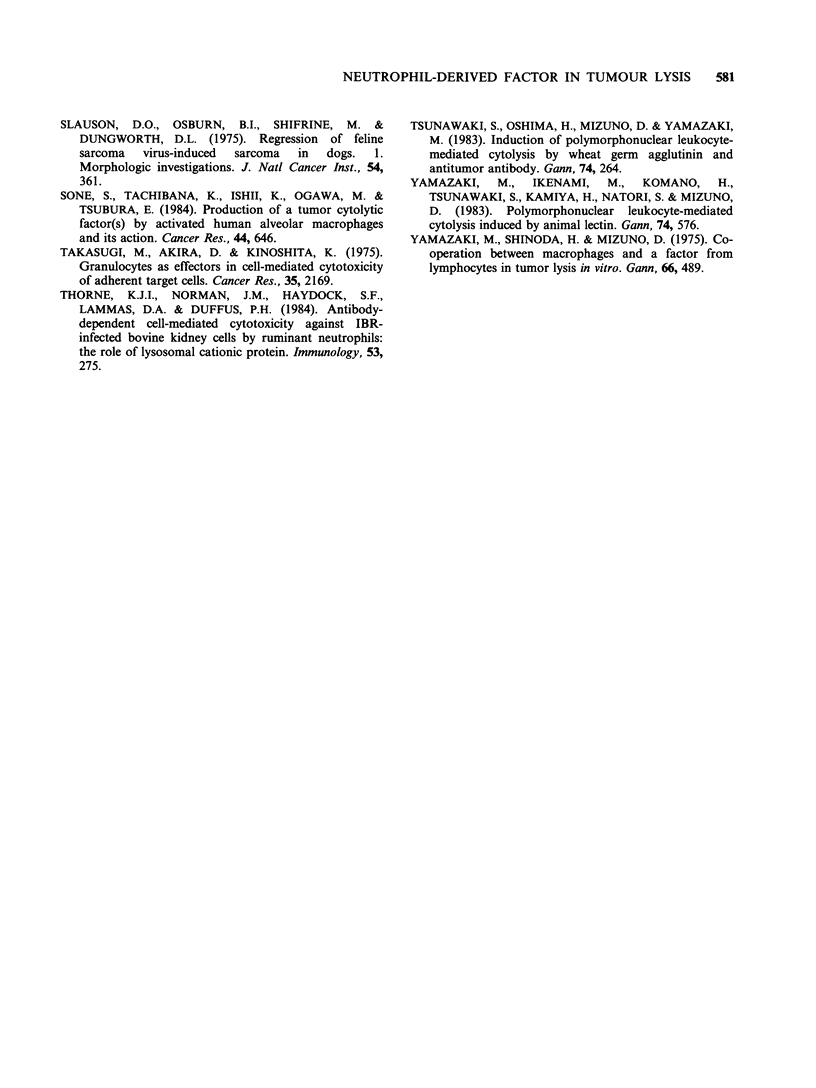


## References

[OCR_00587] Chee D. O., Townsend C. M., Galbraith M. A., Eilber F. R., Morton D. L. (1978). Selective reduction of human tumor cell populations by human granulocytes in vitro.. Cancer Res.

[OCR_00593] Clark R. A., Klebanoff S. J. (1979). Role of the myeloperoxidase-H2O2-halide system in concanavalin A-induced tumor cell killing by human neutrophils.. J Immunol.

[OCR_00599] Dallegri F., Frumento G., Patrone F. (1983). Mechanisms of tumour cell destruction by PMA-activated human neutrophils.. Immunology.

[OCR_00604] Dvorak A. M., Connell A. B., Proppe K., Dvorak H. F. (1978). Immunologic rejection of mammary adenocarcinoma (TA3-St) in C57BL/6 mice: participation of neutrophils and activated macrophages with fibrin formation.. J Immunol.

[OCR_00611] Fisher B., Saffer E. A. (1978). Tumor cells cytotoxicity by granulocytes from peripheral blood of tumor-bearing mice.. J Natl Cancer Inst.

[OCR_00616] Gerrard T. L., Cohen D. J., Kaplan A. M. (1981). Human neutrophil-mediated cytotoxicity to tumor cells.. J Natl Cancer Inst.

[OCR_00621] Godleski J. J., Lee R. E., Leighton J. (1970). Studies on the role of polymorphonuclear leukocytes in neoplastic disease with the chick embryo and Walker carcinosarcoma 256 in vivo and in vitro.. Cancer Res.

[OCR_00633] Ikenami M., Yamazaki M. (1983). [Plant lectin-dependent polymorphonuclear leukocyte-mediated cytolysis].. Yakugaku Zasshi.

[OCR_00639] Korec S., Herberman R. B., Dean J. H., Cannon G. B. (1980). Cytostasis of tumor cell lines by human granulocytes.. Cell Immunol.

[OCR_00644] Kull F. C., Cuatrecasas P. (1984). Necrosin: purification and properties of a cytotoxin derived from a murine macrophage-like cell line.. Proc Natl Acad Sci U S A.

[OCR_00650] Lichtenstein A., Kahle J. (1985). Anti-tumor effect of inflammatory neutrophils: characteristics of in vivo generation and in vitro tumor cell lysis.. Int J Cancer.

[OCR_00656] Morikawa K., Takeda R., Yamazaki M., Mizuno D. (1985). Induction of tumoricidal activity of polymorphonuclear leukocytes by a linear beta-1,3-D-glucan and other immunomodulators in murine cells.. Cancer Res.

[OCR_00670] Nathan C. F., Silverstein S. C., Brukner L. H., Cohn Z. A. (1979). Extracellular cytolysis by activated macrophages and granulocytes. II. Hydrogen peroxide as a mediator of cytotoxicity.. J Exp Med.

[OCR_00676] Pickaver A. H., Ratcliffe N. A., Williams A. E., Smith H. (1972). Cytotoxic effects of peritoneal neutrophils on a syngeneic rat tumour.. Nat New Biol.

[OCR_00682] Ruff M. R., Gifford G. E. (1980). Purification and physico-chemical characterization of rabbit tumor necrosis factor.. J Immunol.

[OCR_00689] Slauson D. O., Osburn B. I., Shifrine M., Dungworth D. L. (1975). Regression of feline sarcoma virus-induced sarcomas in dogs. I. Morphologic investigations.. J Natl Cancer Inst.

[OCR_00696] Sone S., Tachibana K., Ishii K., Ogawara M., Tsubura E. (1984). Production of a tumor cytolytic factor(s) by activated human alveolar macrophages and its action.. Cancer Res.

[OCR_00702] Takasugi M., Akira D., Kinoshita K. (1975). Granulocytes as effectors in cell-mediated cytotoxicity of adherent target cells.. Cancer Res.

[OCR_00707] Thorne K. J., Norman J. M., Haydock S. F., Lammas D. A., Duffus P. H. (1984). Antibody-dependent cell-mediated cytotoxicity against IBR-infected bovine kidney cells by ruminant neutrophils: the role of lysosomal cationic protein.. Immunology.

[OCR_00715] Tsunawaki S., Oshima H., Mizuno D., Yamazaki M. (1983). Induction of polymorphonuclear leukocyte-mediated cytolysis by wheat germ agglutinin and antitumor antibody.. Gan.

[OCR_00721] Yamazaki M., Ikenami M., Komano H., Tsunawaki S., Kamiya H., Natori S., Mizuno D. (1983). Polymorphonuclear leukocyte-mediated cytolysis induced by animal lectin.. Gan.

[OCR_00727] Yamazaki M., Shinoda H., Mizuno D. (1975). Co-operation between macrophages and a factor from lymphocytes in tumor lysis in vitro.. Gan.

